# Modification of Liposomal Properties by an Engineered Gemini Surfactant

**DOI:** 10.1021/acs.langmuir.4c03043

**Published:** 2025-01-25

**Authors:** Ala’a F. Eftaiha, Buti Suryabrahmam, Nicholas B. Morris, Abdussalam K. Qaroush, Khaleel I. Assaf, Dina M. Foudeh, Suhad B. Hammad, Rana Ashkar

**Affiliations:** † Department of Chemistry, Faculty of Science, The Hashemite University, Zarqa 13133, Jordan; ‡ Department of Physics, Virginia Tech, Blacksburg, Virginia 24061, United States; § Center for Soft Matter and Biological Physics, Virginia Tech, Blacksburg, Virginia 24061, United States; ∥ Department of Chemistry, Faculty of Science, The University of Jordan, Amman 11942, Jordan; ⊥ Department of Chemistry, Faculty of Science, Al-Balqa Applied University, Al-Salt 19117, Jordan; # Macromolecular Innovation Institute, Virginia Tech, Blacksburg, Virginia 24061, United States

## Abstract

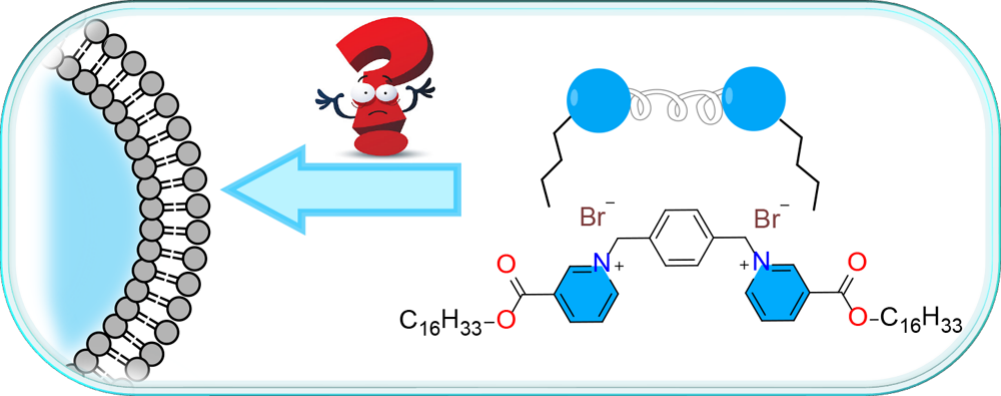

Lipid membranes form the primary structure of cell membranes and serve as configurable interfaces across numerous applications including biosensing technologies, antifungal treatments, and therapeutic platforms. Therefore, the modification of lipid membranes by additives has important consequences in both biological processes and practical applications. In this study, we investigated a nicotinic-acid–based gemini surfactant (NAGS) as a chemically tunable molecular additive for modulating the structure and phase behavior of liposomal membranes. We specifically focused on NAGS with hydrocarbon chains that mirror those of lipid molecules. By introducing NAGS to phosphatidylcholine membranes with lipids of identical and varied chain lengths or degrees of unsaturation, we demonstrated the effects of headgroup interactions and chain mismatch between NAGS and membrane lipids. Using small-angle X-ray scattering, we showed that regardless of chain compatibility or mismatch, NAGS reduced the thickness and packing density of fluid lipid membranes. Further observations by fluorescence microscopy revealed the emergence of ordered-disordered domains upon cooling to room temperature. The observed phases were quite distinct from those of lipid membranes with analogous chain compositions, emphasizing the importance of NAGS headgroup chemistry in mediating domain formation and stabilization. These findings open new possibilities for exploiting NAGS in tuning the structure and organization of liposomal membranes with potential applications in drug delivery and biomedical imaging.

## Introduction

Lipid bilayers form the primary structural matrix of cell membranes, creating a protective barrier that maintains cellular integrity and viability. Inspired by biological functions, lipid bilayers have also been utilized in numerous practical applications, including vaccine delivery and biosensing technologies. As such, controlled modulations of lipid bilayer properties have important consequences in biological processes, therapeutics, and biotechnologies. Applications range from artificial cells to antibacterial and antifungal treatments.^1−5^ For example, the disruption of lipid membranes by additive molecules could provide alternate solutions to antimicrobial resistance, a growing threat to global public health. In addition, tuning bilayer properties is often needed in the design of liposomal nanoparticles as effective nanocarriers for active compounds with applications in cosmetics, pharmaceuticals, and artificial cell technologies.^6−8^ The introduction of additives can have profound effects on the bilayer properties, potentially leading to the development of “*smart*”, configurable, and adaptive liposomes.^8^ These effects include alterations of membrane thickness and reorganization, biocompatibility, and lipid–protein interactions.^9,10^ The use of additives in controlling the phase separation in lipid membranes holds tremendous potential for next-generation multifunctional liposomes or synthetic cells.^11−13^ Such control over the spatial distribution of molecular components enables nano- and micropatterning for advanced site-specific functionality.^14,15^

**Scheme 1:**
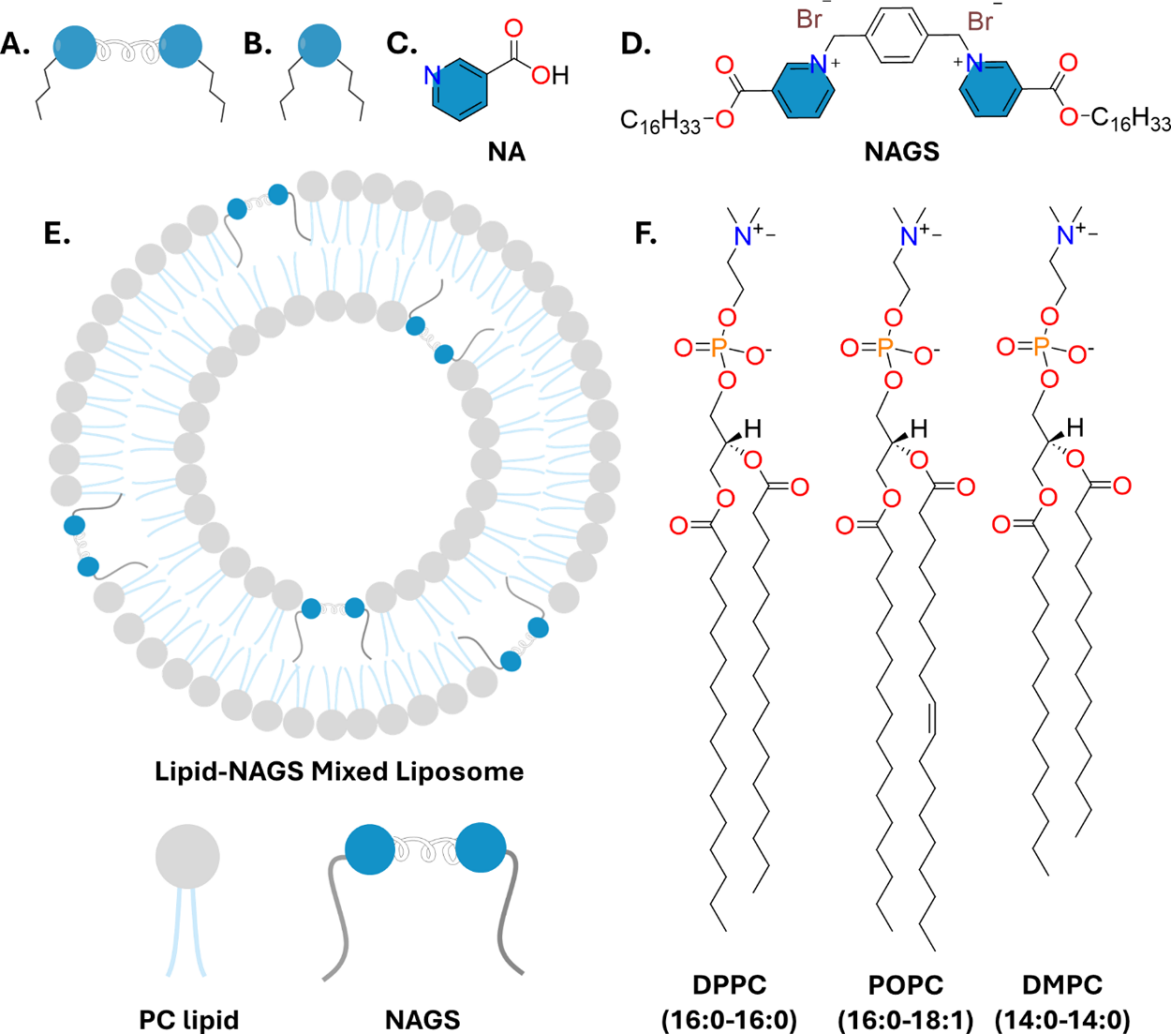
Chemical Structures and Schematic Representations of Gemini Surfactants and Phospholipids Cartoons of (A) gemini surfactant molecule consisting of two amphiphilic hydrocarbon chains linked through a tunable linker at the head group level and (B) phospholipid molecule. The curly ribbon and zigzag lines denote the organic linker and the hydrocarbon chains, respectively. The blue circle represents the head group; (C, D) Chemical structure of nicotinic acid (NA); 1,1′-(1,4-phenylenebis(methylene))bis(3-((hexadecyloxy)carbonyl)pyridin-1-ium) bromide (NAGS); (E) Cartoon of a lipid-NAGS mixed liposome; (F) Chemical structure of 1,2-dipalmitoyl-*sn*-glycero-3-phosphocholine (DPPC, diC16:0); 1,2-dimyristoyl-*sn*-glycero-3-phosphocholine (DMPC, diC14:0); and 1-palmitoyl-2-oleoyl-*sn*-glycero-3-phosphocholine (POPC, C16:0-C18:1).

One approach to fine-tune bilayer structure, organization, and overall functionality involves the use of gemini surfactants (GSs) (see Scheme 1A).^16−24^ While GSs are typically viewed as additives, their molecular architecture represents a hybrid lipid molecule (Scheme 1B) thus enabling their partitioning into lipid membranes and the potential regulation of various membrane-associated processes.^25^ The dimeric character and inherent permanent charge of GSs confer exceptional properties as surface modifiers, antimicrobial agents, gene carriers, and drug delivery systems.^26−28^ For instance, cationic GSs have the potential to serve as nonviral vectors for the delivery of genetic material, including DNA and RNA.^29−33^ This could mark a significant milestone in addressing the inherent limitations of lipid nanocarriers^34^ as agents in protecting and delivering mRNA, e.g., COVID-19 vaccines.^35^ Importantly, the structural similarity between GSs and phospholipids combined with the tunability of their chain length and linker chemistry provides unconventional pathways for their integration into lipid membranes and their use as molecular regulators for synthetic or biological membranes.

Utilizing naturally occurring materials will improve the suitability of GSs for biomedical applications. Nicotinic acid (NA) (Scheme 1C), also known as niacin or vitamin B3, has been used to synthesize biorenewable pyridinium-GSs.^36−40^ The use of such materials offers numerous advantages over traditional petrochemical-based sources and holds promise for diverse applications across various industries including pharmaceuticals, dietary supplements, and personal care products.^41^ While ammonium-based GSs have dominated the landscape of cationic GSs due to their facile synthesis and purification,^42^ pyridinium-based GSs have emerged as promising alternatives as they exhibit notably lower critical micelle concentrations, a characteristic often attributed to their reduced conformational flexibility.^43^ Furthermore, the presence of ester linkages between the surfactant headgroup and chains, as in lipid structures, enhances the biodegradability of NA-derived surfactants. Similar to pyridinium cations bearing ester side chains, these compounds are expected to exhibit high biodegradability under aerobic conditions.^44^

We have recently reported the synthesis of a series of GSs using NA tethered with rigid (*p*-xylenylene) and flexible (ethylene and butylene) spacers.^45,46^ Our earlier studies have shown that nicotinic acid gemini surfactants (NAGS) (Scheme 1D) form stable Langmuir monolayers and exhibit a strong tendency to self-assemble into well-defined, discrete domains at air–water and air–solid interfaces. The mixing thermodynamics of NAGS with dipalmitoylphosphatidylcholine (DPPC) showed a decrease in the Gibbs free energy of mixing upon monolayer compression, suggesting strong affinity and compatibility between NAGS and DPPC lipids.^45^

In this study, we explored how NAGS with two hexadecyl carbon chains (C_16_H_33_−) (Scheme 1D) influence the thermodynamic and structural properties of lipid bilayers with matching or contrasting acyl chains (Scheme 1E). Specifically, we examined three phosphatidylcholine lipids DPPC (diC16:0), POPC (C16:0-C18:1), and DMPC (diC14:0) where *X*:*Y,* respectively, represent the number of carbons and double bonds in each of the lipid acyl chains (Scheme 1F). In the case of DPPC, the tail–tail mismatch is minimized due to the structural similarity between NAGS and DPPC hydrocarbon chains, enabling focused studies on the effect of headgroup variations. On the other hand, the use of POPC and DMPC enabled investigations of the influence of chain unsaturation and length mismatch. To obtain insights into lipid-NAGS interactions, we conducted molecular modeling to evaluate the pair interaction energies of lipid-NAGS complexes. Additionally, we utilized small-angle X-ray scattering (SAXS) to examine the effects of NAGS-lipid interactions on membrane thickness and molecular packing, differential scanning calorimetry (DSC) to determine the phase behavior of NAGS-lipid mixtures, and epifluorescence microscopy to investigate emergent phase-separated structures. SAXS and DSC studies were performed on small unilamellar vesicles (SUVs), while epifluorescence studies were performed on giant unilamellar vesicles (GUVs).

## Materials and Methods

### Materials

1,2-Dipalmitoyl-*sn*-glycero-3-phosphocholine (DPPC), 1-palmitoyl-2-oleoyl-*sn*-glycero-3-phosphocholine (POPC), 1,2-dimyristoyl-*sn*-glycero-3-phosphocholine (DMPC), and 1-palmitoyl-2-oleoyl-*sn*-glycero-3-phospho-l-serine (POPS) were purchased from Avanti Polar Lipid. All lipids were used as received without further purification. Chloroform (Biotech. Grade, ≥ 99.8%) and ethanol (200 Proof, 100% absolute) were obtained from Sigma-Aldrich and Decon Laboratories, Inc., respectively. Indium tin oxide (ITO) coated glass plates were purchased from Techinstro. 1,1′-Didodecyl-3,3,3′,3′-tetramethylindocarbocyanine perchlorate (DiI-C12) and sucrose were purchased from Invitrogen and Sigma-Aldrich, respectively. NAGS was synthesized according to our previously published protocol.^45^

### Preparation of Small Unilamellar Vesicles (SUVs)

Suspensions of SUVs were prepared at 20 mg/mL by weighing the required amounts of lipids or lipid-NAGS mixtures with NAGS content of 10–50 mol %. All samples contained 4 mol % of POPS to ensure liposomal unilamellarity.^47^ After weighing, the lipids or lipid-NAGS mixtures were dissolved in chloroform and then dried under a stream of argon gas before drying overnight under vacuum at 35 °C to remove any residual solvent. The samples were then hydrated with filtered Milli-Q water at 60 °C and vortexed until homogeneously mixed. Each sample underwent five consecutive freeze/thaw cycles at −80 and 60 °C for ∼4 min each, followed by incubation at 60 °C for 3 h. DSC measurements indicated that this incubation period was essential to ensuring the partitioning of NAGS into lipid membranes. Without this incubation, the thermogram of any mixture was identical to that of the pristine lipid. Each suspension was then subjected to 30 consecutive extrusion cycles by passing it back and forth through a polycarbonate filter with 100nm-diamater pores using a temperature-controlled home-built automated extruder.^48^ All extrusions in this study were performed at a rate of 1 mL/min and at a temperature of 65 °C. For SAXS measurements, the concentration of the extruded sample was maintained at 20 mg/mL. For DSC measurements, the suspensions were diluted to 10 mg/mL using filtered Milli-Q water at 60 °C. We note that NAGS-containing mixtures were marked by a yellow (or yellowish) color, attributed to the formation of a charge transfer complex. A detailed discussion of this phenomenon is provided in the Supporting Information (SI).

### Preparation of Giant Unilamellar Vesicles (GUVs)

GUVs were prepared by the electroformation method using indium tin oxide (ITO) coated glass plates.^49,50^ The electroformation chamber was made by pasting a 2 mm thick and 22 mm diameter Teflon spacer. Initially, 7–10 μL of a 1 mg/mL SUV solution was coated at the center of the Teflon spacer and dried with argon gas. Within 10 min, the Teflon spacer was filled with preheated 1.5 mL of 100 mM sucrose solution. Another ITO plate was placed on the Teflon spacer to complete the electroformation chamber. The GUVs were grown for 90 min by applying AC square wave with a frequency of 12 Hz and voltage of 1.2 V_rms_.^51^ To maintain the membrane fluidity during GUV growth, the ITO plates were placed on a hot plate set to 10 °C above the main phase transition temperature of the lipid used lipid. All NAGS-containing GUVs were prepared using 30 mol % NAGS, and 2 mol % of DiI-C12 dye was added for visualization of phase separation.

### Dynamic Light Scattering (DLS) Measurements

The particle size and size distribution of SUVs were measured by using a Malvern Zetasizer Nano ZS. Liposomal suspensions were measured at an initial concentration of 1 mg/mL. Successive dilutions using filtered Milli-Q water were performed until a good-quality autocorrelation function was obtained. Samples were allowed to equilibrate at 25 °C before conducting measurements using a refractive index of 1.48 and viscosity of 0.89 mPa·s. The hydrodynamic radius (*R*_H_) was estimated from the translational diffusion coefficient (*D*) using the Stokes–Einstein equation as follows: *D* = *k*_B_*T*/6πη*R*_H_, where *k*_B_ is the Boltzmann constant, *T* is the absolute temperature, and η is the solution viscosity.

### Differential Scanning Calorimetry (DSC) Measurements

Cooling/heating thermograms of freshly prepared SUVs were recorded on a TA Instruments Nano DSC. Measurements were conducted at 3 atm, with a scanning rate of 0.2 °C/min, between 10 and 60 °C using 300 μL of approximately 10 mg/mL liposomal suspensions. Five cycles were conducted to validate the reproducibility, and the results of the first cooling and heating scans were reported. Samples were equilibrated for 10 min before the start of the measurement and after each cycle to ensure temperature uniformity throughout the sample, prevent artifacts, and minimize hysteresis effects that might arise from slow temperature response.

### Small Angle X-ray Scattering (SAXS) Measurements

SAXS measurements were performed on a Xenocs Xeuss 3.0 instrument with a Dectris Eiger2 4M detector using a CuKα source. For each sample, 100 μL of 20 mg/mL liposomal suspensions were loaded into 1.5 mm diameter borosilicate capillaries and placed onto a Peltier stage set at 25 or 50 °C and left to equilibrate for 20 min. A sample-to-detector distance was selected to obtain a *q-*range from 0.01 to 0.8 Å^–1^ where the scattering wavevector transfer *q* is defined as, , such that λ is the X-ray wavelength and θ is the scattering angle relative to the incident beam. Eight consecutive runs of 30 min were collected per sample and were merged and finally background-subtracted from a sample containing only water. Data were analyzed using the *SasView* software^52^ and modeled by the theoretical framework described in the SI. This framework was adapted from Lewis-Laurent et al. using a 5-shell spherical model accounting for the two headgroup layers, the acyl chain layer from the two opposing leaflets, and the interleaflet region at the membrane midplane.^53^ This allowed for the reconstruction of the electron density (ED) profiles from the SAXS data fits. Further details of the scattering model and data fitting can be found in the SI.

### Computational Methodology for Assessing Lipid-NAGS Interactions

The initial structure of the free compounds (i.e., lipids and NAGS) was constructed based on standard bond distances and angles, and energy minimization was conducted using molecular modeling by employing the AMBER force field^54^ and conjugate gradient algorithm HyperChem^55^ (release 8.0.10 professional, Hypercube Inc.). The interaction energy between NAGS and each of the lipids was calculated using the semiempirical PM3 method, relative to free molecules in the gas phase using Gaussian 09 software.^56^

### Fluorescence Microscopy Measurements

The emergent phase separation of lipid-NAGS membranes was measured on GUVs by employing fluorescence microscopy using a DiI-C12 dye. This dye is known to preferentially partition into disordered lipid domains.^57^ To ensure homogeneity in membrane composition across formed GUVs, samples were prepared at temperatures higher than the main phase transition temperature of the membranes, as determined by DSC. After GUV preparation, the electroformation chamber was directly transferred onto the microscope stage and GUVs were observed as they cooled down to room temperature. The GUVs were observed by epifluorescence imaging using an IX73 inverted microscope. Images were obtained and analyzed using ImageJ software.^58^

## Results and Discussion

### NAGS Partitioning into Liposomes with Identical-Chain Lipids

The incorporation of NAGS into lipid membranes is necessary for their use as membrane additives. Our dynamic light scattering (DLS) measurements confirmed the formation of mixed lipid-NAGS liposomes, as described in the SI. More importantly, the integration of NAGS into the liposomes was evident in differential scanning calorimetry (DSC) results and was further validated by our molecular modeling. As indicated earlier, the incubation of NAGS-lipid suspensions was necessary for NAGS partitioning into liposomal membranes and subsequent changes in the measured thermograms.

**Figure 1:**
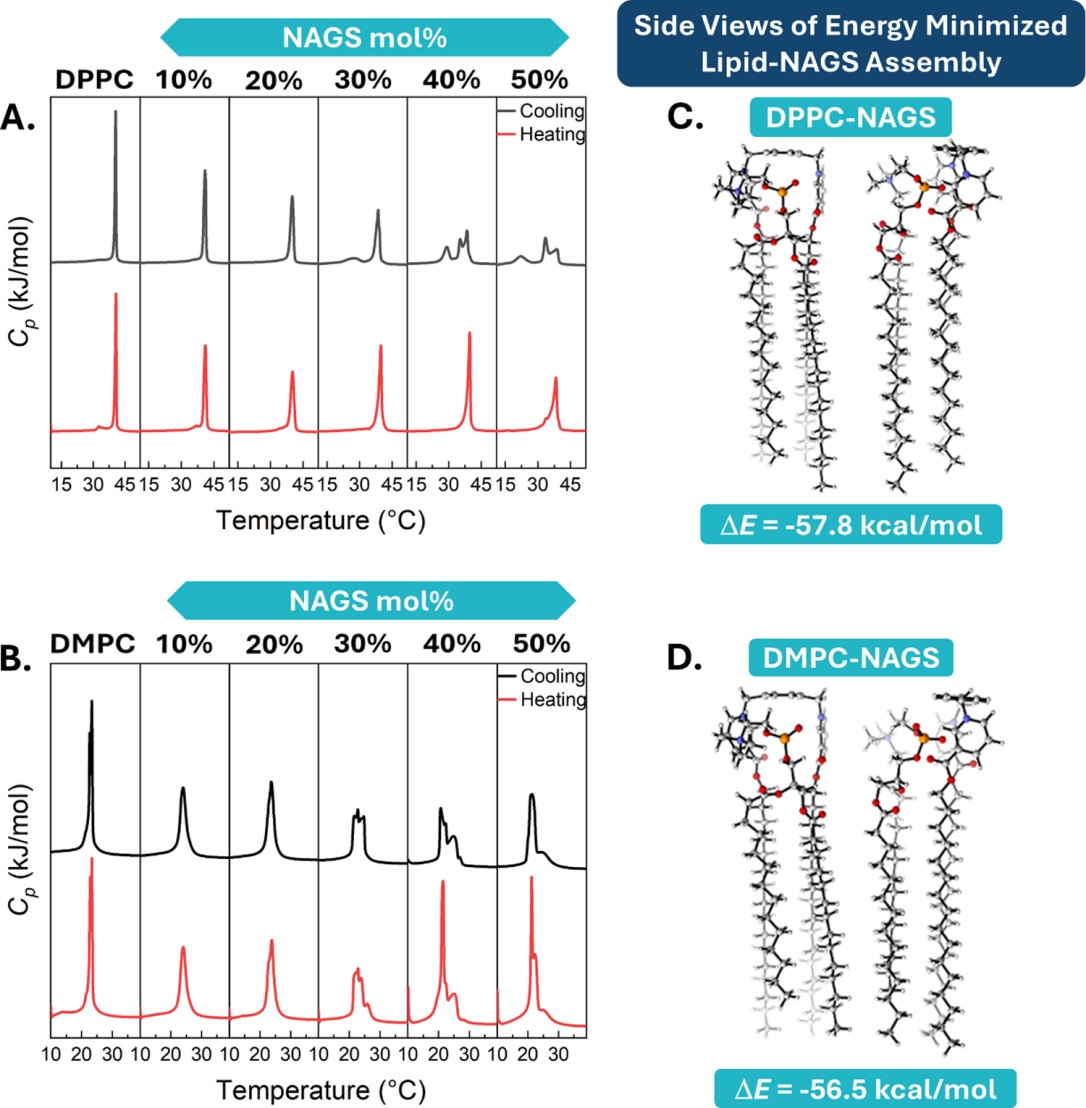
DSC thermograms (cooling: black trace; heating: red trace) and side views of energy minimized pairs and the corresponding interaction energy of (A) DPPC-NAGS and (B) DMPC-NAGS. For DSC cooling thermograms, samples were rapidly preheated to 60 °C and subsequently cooled to 10 °C. All samples were doped with 4 mol % of POPS. At and above 30 mol %, NAGS induce multiple phase transitions upon cooling, suggesting strong interactions between NAGS and PC lipids, leading to complex phase behavior. These observations are consistent with the semiempirical calculations of interaction energies between NAGS and PC lipids reported in (C) and (D). (C, D) Two side views of DPPC and DMPC complexes with NAGS, respectively, based on energy minimization calculations. The obtained structures show a strong affinity between the NAGS positively charged pyridinium rings and the lipid negatively charged phosphate group. Similar structures were obtained for POPC-NAGS pairs (not shown).

We first investigated the effects of NAGS on DPPC membranes with almost identical chains to illustrate the influence of the NAGS headgroup chemistry on membrane properties. As shown in Figure 1, the cooling thermogram of preheated DPPC SUVs showed a sharp peak at 40.5 °C, characteristic of the DPPC phase transition from liquid to gel phase. A similar cooling thermogram was observed for mixtures containing 10 and 20 mol % of NAGS, with a slight shift in the main liquid-to-gel transition temperature. The cooling thermograms for the 30–50 mol % NAGS mixtures displayed multiple peaks, indicating a more complex phase behavior compared to the 10 and 20 mol % mixtures. Specifically, the liposomal DPPC membranes with 30 mol % NAGS exhibited two peaks, with shifts to lower temperatures indicating membrane fluidization by NAGS. Increasing NAGS concentration between 40 and 50 mol % yielded notable alterations in the cooling thermograms. The splitting of the high-temperature peak into two separate peaks suggests the coexistence of phospholipid-rich and mixed DPPC-NAGS domains. Here it is important to point out that NAGS-only suspensions did not exhibit any transitions over the investigated temperature range. Therefore, the emergent peaks at higher NAGS concentrations cannot be attributed to the transition temperature of NAGS but are rather indicative of mixed lipid-NAGS phases. These observations suggest a heterogeneous distribution of NAGS within DPPC membranes with increasing NAGS concentrations, as shown later with microscopy.

In contrast to the cooling cycles, the heating thermograms of all mixtures showed a single main transition peak. Interestingly, these peaks coincided with those obtained in cooling thermograms for the 10 and 20 mol % DPPC-NAGS mixtures, indicating cooperative melting or gelling behavior at relatively low NAGS concentrations. On the other hand, the observed hysteresis between the cooling and heating thermograms for higher NAGS concentrations (30–50 mol %) could be attributed to different phase-separation kinetics induced by NAGS. During the cooling process, the high thermal energy and inherently rapid kinetics lead to complex phase behavior characterized by multiple peaks. However, upon heating, the transition from a gel phase introduces kinetic constraints, resulting in a single peak limiting the system’s ability to exhibit the same degree of complexity observed during cooling. Interestingly, the pretransition peak in DPPC membranes, typical of the rippled gel phase, persisted up to NAGS concentrations of 10 mol % but got suppressed at higher NAGS content. This indicates that the addition of NAGS disrupts the highly ordered molecular arrangements in the DPPC gel phase, resulting in a less ordered lipid assembly. While both DPPC and NAGS have palmitic chains, the repulsive electrostatic interactions between NAGS headgroups favor lower packing densities, consistent with our other observations.

To better understand the thermal behavior of NAGS within DPPC liposomes, we consider the substructures of NAGS, namely, hexadecyl nicotinate and palmitic acid, and their individual effects on DPPC membranes. Here we note that special attention must be paid to the charged ring structure of NAGS, which plays a crucial role in its interaction with the negatively charged phosphate group of DPPC. For instance, hexadecyl nicotinate, a simplified version of NAGS lacking the linker, has been shown in previous studies to exhibit complete miscibility with DPPC up to 50 mol %.^36^ By further inspecting the plamitic acid moeity of hexadecyl nicotinate, i.e., NAGS chains, previous studies have revealed an increase in the transition temperature of DPPC bilayers upon the incorporation of 40 mol % palmitic acid.^59^ These findings suggest that the unique chemical structure of NAGS, including the presence of the linker, results in the formation of coexisting domains in the form of phospholipid-rich regions and mixed DPPC-NAGS domains at high NAGS concentrations.

### Effects of NAGS on Phase Transitions in Liposomal Membranes with Chain Mismatch

To further investigate the effects of chain mismatch between NAGS and lipids, we performed DSC studies on DMPC-NAGS liposomes, where DMPC chains are two carbons shorter than the NAGS chains. Our measurements on DMPC liposomes revealed the presence of two split adjacent peaks at approximately 22.7 and 23.3 °C (Figure 1), in agreement with previous measurements reported by Ebel et al.^60^ In comparison, DMPC multilamellar vesicles (MLVs) have been shown to exhibit a single prominent peak.^61^ These differences may be attributed to chain density fluctuations in the plane of the bilayer that are enhanced by increasing curvature leading to peak broadening and splitting.^62^

Upon the introduction of NAGS up to 20 mol %, a notable change occurred as evidenced by the appearance of a single peak in both the cooling and heating thermograms. Samples containing 30 mol % NAGS displayed a triple peak structure. When compared to the corresponding DPPC system (Figure 1), the presence of a multipeak at a 30% mole fraction signifies the role of head–head interaction in the phase behavior of lipid-NAGS membranes, as illustrated later with microscopy. Moreover, as we increased the NAGS concentration, a trend toward more complex, multipeaked patterns became apparent, reflecting a more heterogeneous molecular environment within the membrane. The observation of a similar set of peaks upon cooling and heating reveals minimal hysteresis in DMPC membranes, indicating a lack of significant kinetic constraints unlike what we observed in DPPC-NAGS mixtures. These differences might arise from the lower melting temperature of DMPC and the fact that the lowest equilibration temperature was much closer to the main transition temperature of DMPC than that of DPPC, thus not allowing the DMPC-NAGS membranes to transition deep into the gel phase.

Notably, a previous study on dipalmitoylphosphatidylethanolamine (DPPE) membranes at low scan rates has shown a similar single-peak transition during heating cycles but multiple-peak transitions during cooling cycles.^63^ Utilizing a combination of DSC and time-resolved X-ray diffraction, the study showed that the multipeak pattern became distinct with an increased incubation time in the liquid-crystalline phase. It concluded that these peaks represented a mixture of domains that differed in thermal behavior, similar to our observations and conclusions.

### Molecular Mechanisms of Lipid-NAGS Interactions

To gain mechanistic insights into the interactions between lipids and the GS, we evaluated the energy of each pair using a semiempirical method relative to free molecules in the gas phase. These calculations should be approached cautiously when applied to liposomes, as they overlook significant factors such as hydration and interactions with neighboring molecules but still can offer qualitative insights. The side views of the energy-minimized structures of NAGS paired with DPPC and DMPC are illustrated in Figure 1C,D. The observed lipid-NAGS pair geometry suggests that the hydrophobic chains are aligned in a parallel manner, facilitating attractive van der Waals interactions. For example, both DPPC and DMPC exhibit assembly patterns similar to those of NAGS, as shown in Figure 1. On the other hand, we observed that headgroup association in lipid-NAGS pairs was facilitated by Coulombic attractions between the negatively charged phosphate group of PC lipids and the positively charged pyridinium ring of NAGS.

Importantly, we found that the interaction between lipid-NAGS pairs is more favorable than that between lipid–lipid and NAGS–NAGS pairs. The strongest interaction energy (Δ*E*) was obtained for the DPPC-NAGS pair, measuring −57.8 kcal/mol, followed by DMPC-NAGS and POPC-NAGS, with Δ*E* of −56.5 kcal/mol and −51.0 kcal/mol, respectively. To contextualize these results, we note that the interaction energies for lipid–lipid pairs were found to be Δ*E* = −34.4 kcal/mol for DPPC, −24.2 kcal/mol for DMPC, and −21.8 kcal/mol for POPC lipid pairs. In comparison, Δ*E* for the NAGS–NAGS pair was calculated to be ∼132 kcal/mol, indicating highly repulsive interaction. Recalling that all lipid-NAGS pairs or lipid–lipid pairs have identical head–head interactions, we attribute the observed differences in Δ*E* with different lipids to chain variations, including chain length and unsaturation. Notably, POPC (16:0–18:1) which has an unsaturated chain exhibits the weakest interaction energy, consistent with the effects of unsaturation on chain conformations.

### NAGS-Induced Thinning and Dilation of Fluid Lipid Membranes

**Figure 2:**
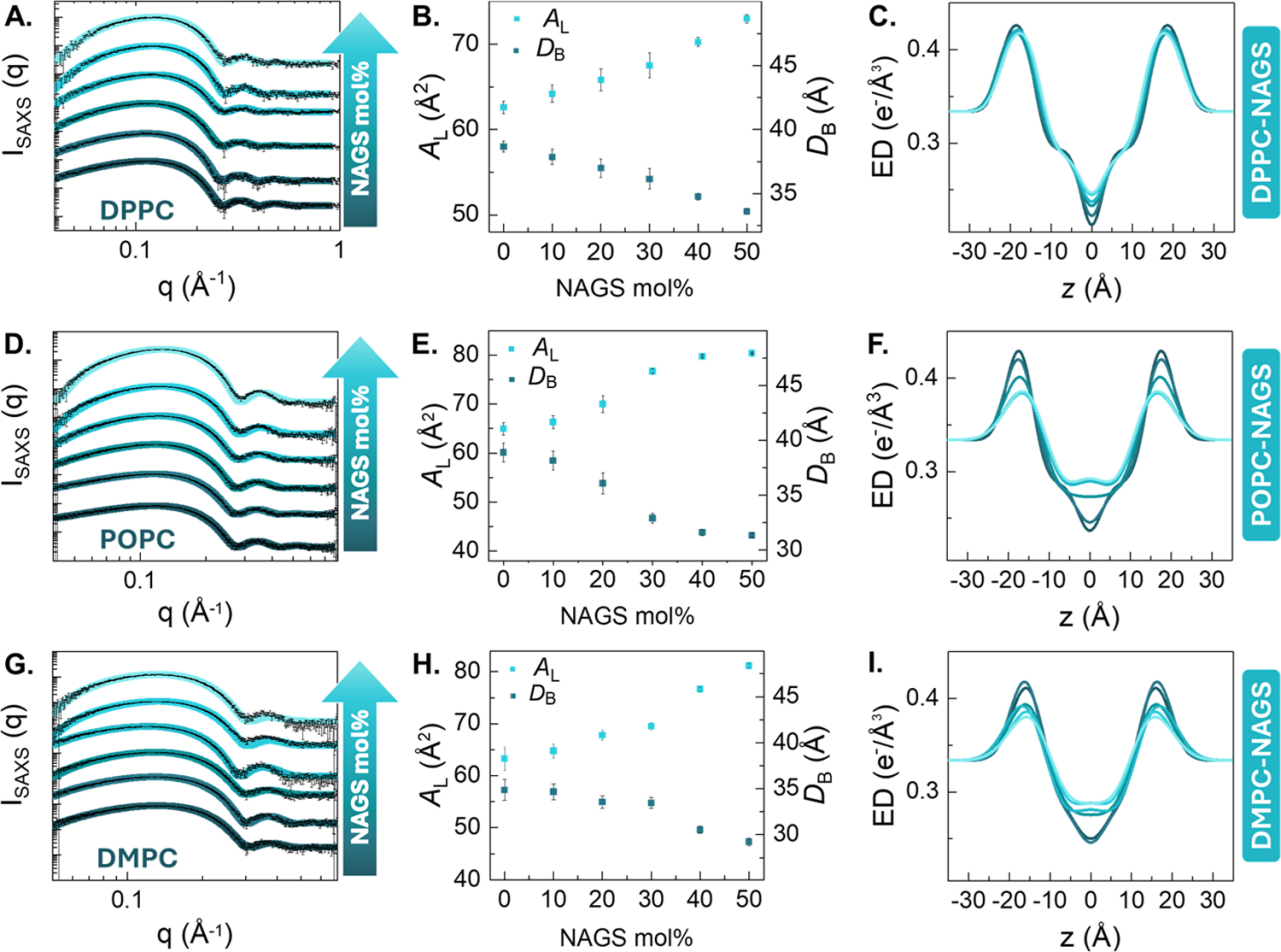
(A, D, G) Scattering intensity profiles plotted as a function of the scattering wavevector transfer, *q*, for SUVs composed of DPPC-NAGS, POPC-NAGS, and DMPC-NAGS measured at 50 °C. The NAGS content varied from 0 to 50 mol % in increments of 10 mol %; (B, E, H) area per lipid (*A*_L_) and membrane thickness (*D*_B_) were obtained from SAXS data analysis for DPPC-NAGS, POPC-NAGS, and DMPC-NAGS, respectively. (C, F, I) Electron density (ED) profiles were reconstructed from fitted model parameters along the membrane normal for DPPC-NAGS, POPC-NAGS, and DMPC-NAGS, respectively. The ED increase at *z* = 0 can be ascribed to increased interdigitation of the terminal methyl groups of the chains. Head group volumes together with electron count in the two hydrocarbon chains and the head groups are shown in Table S2. The SAXS fitting parameters are shown in Tables S3–S5.

The membrane structure of pure liposomes and NAGS-containing liposomes were investigated using SAXS.^64^ The obtained scattering intensities are shown in Figure 2 for NAGS concentrations ranging from 0 to 50 mol %, in steps of 10 mol %. Data fitting using the five-shell model^65^ described in the SI yielded the average area per lipid (*A*_L_) and the total bilayer thickness (*D*_B_). The fits also enabled the determination of the electron density (ED) profile of all of the studied membranes.

For DPPC membranes (Figure 2A), the addition of NAGS resulted in shifts of the scattering minima to higher *q* values, typically associated with a decrease in membrane thickness. This was validated by the fitted parameters (Figure 2B), showing a decrease in *D*_B_ and a corresponding increase in *A*_L_ with an increasing NAGS concentration. Interestingly, the ED profiles showed that the addition of NAGS changed the methyl group density in the bilayer center, further illustrating the structural effects of NAGS on the DPPC membranes (Figure 2C). While these findings indicate that the presence of NAGS induces alterations in the structure of DPPC membranes, the structural integrity of the liposomes remains preserved even at high NAGS concentrations.

POPC-NAGS membranes exhibited similar trends (Figure 2D), also signifying membrane thinning and an increase in *A*_L_ (Figure 2E). Importantly, the changes in membrane thickness in POPC-NAGS membranes were much more pronounced than in DPPC-NAGS membranes, leading to significant changes in the obtained ED profiles, especially in the bilayer center (see Figure 2F). Studies on DMPC-NAGS membranes resulted in similar behavior (Figure 2G–I), indicating similar effects of NAGS on the DMPC membrane structure. Furthermore, the reconstructed profiles showed an increase in ED at the center of the bilayer, which can be attributed to the interdigitation of the chains from the two opposing leaflets induced by NAGS disruption of the packing at the headgroup region. This effect was minimal for DPPC since the chains were matched to the surfactant, but it was more pronounced in DMPC-NAGS and POPC-NAGS membranes with considerable chain mismatch.

At the highest NAGS concentration (50 mol %), *A*_L_ increased by 28, 24, and 17%, while *D*_B_ decreased by 16, 19, and 13% for DMPC, POPC, and DPPC liposomes, respectively. These results confirm our hypothesis that chain unsaturation and length mismatch have relatively more influence on PC-membrane thickness and molecular packing, particularly at high NAGS content when compared to those of DPPC-NAGS mixed liposomes.

Using molecular dynamics simulations, Peters et al. reported that stearate and oleate (charged saturated and unsaturated fatty acids, respectively) induce interdigitation of the acyl chains in DMPC bilayers, leading to membrane thinning. This might be correlated with the effect of NAGS, which has charged headgroups and long hydrocarbon chains.^66^ Our results are also consistent with earlier studies of ammonium-based GSs reporting similar conclusions regarding the effects of GS on molecular packing, albeit based on indirect deductions from DSC measurements as opposed to direct structural observations.^17,67^

To elucidate the underlying mechanisms and physicochemical interactions governing NAGS-membrane interactions, we compare the molecular structures of NAGS to those of its ammonium GS analogues. The amphiphilic structure of NAGS, comprising hydrophilic pyridinium headgroups and hydrophobic hydrocarbon tails, significantly impacts its liposomal interactions. The balance between these moieties and the electrostatic attraction between NAGS cationic pyridinium groups and the lipid anionic phosphate group determines the extent of NAGS integration and organization within the lipid bilayer. Variations in GS polar head groups, spacer rigidity, and tail length—coupled with their lipid compatibility—can result in different interactions at the water interface and within the bilayer core.

For example, previous studies have demonstrated changes in bilayer thickness with bis-quaternary ammonium surfactants (notated as *m–s–m*, where “*m*” represents the number of carbon atoms in the tails and “*s*” represents the number of carbon atoms in the linker). This was indirectly inferred from fluorescence and DSC measurements, which revealed a reduction in the overall structural order of DPPC with *12–2–12* surfactants or the formation of new ordered structures with *16–2–16* and *18–2–18* surfactants.^17^ Additionally, X-ray diffraction studies on fluid lamellar egg yolk phosphatidylcholine bilayers incorporating *m–4–m* surfactants (*m* = 7–16) showed a decrease in bilayer thickness and an increase in lipid surface area, with the maximum effect observed at an alkyl chain length of *m* = 8.^68^ However, we point out that the reduced conformational flexibility of the pyridinium moiety in NAGS^43^ offers new possibilities that are distinct from their commonly used ammonium analogues.

To further separate out the effects of charge on membrane properties, future studies can take advantage of Zeta potential measurements. Previous investigations have demonstrated that headgroup charge can have significant effects on lipid packing, membrane thickness, and other physical membrane properties (e.g., bending rigidity),^69,70^ all of which are crucial for NAGS applications in liposomal technologies and biological applications.^71^

### NAGS Stabilization of Ordered Phases in Lipid Membranes

**Figure 3:**
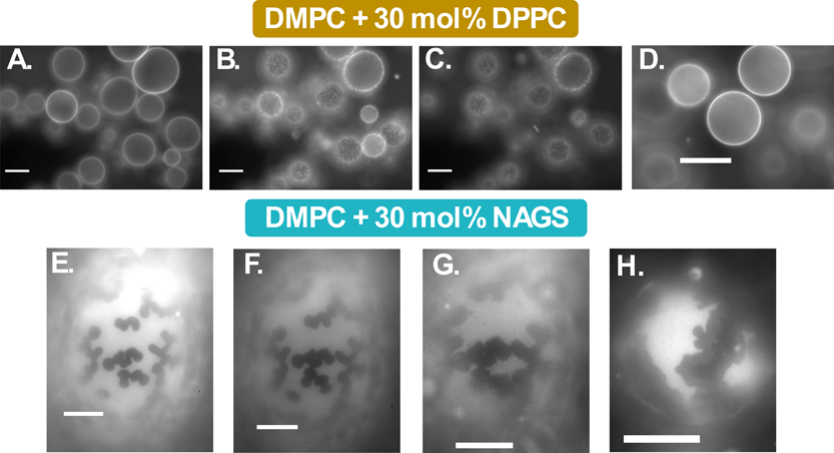
Epifluorescence images of: (A–D) DMPC-30 mol % DPPC GUVs measured at 55, 32, 28, and 23 °C, respectively; (E–H) DMPC-30 mol % NAGS GUVs (measured at 30, 28, 26, and 23 °C, respectively). Samples were doped with the DiI-C12 dye, prepared at high temperature, and observed as they cooled to room temperature. The scale bar in all images is 20 μm.

To determine the effects of NAGS on membrane patterning, we investigated the phase behavior of DPPC, DMPC, and POPC GUVs with 30 mol % NAGS with decreasing temperature using epifluorescence microscopy. As a control experiment, we studied GUVs composed of DMPC with 30 mol % DPPC; i.e. lipids with identical headgroups and mismatched chains, with well-known phase behavior.^72,73^ In these studies, we tracked the formation and growth of ordered domains as the sample was cooled from 55 °C to room temperature. As shown in Figure 3A, a uniform phase was observed above the upper transition temperature (i.e., ∼41 °C), as expected. Dark domains with striped appearances emerged at 32 °C, Figure 3B. These domains could be attributed to the DPPC-rich ripple phase,^73^ which closely aligns with the first peak in the DSC cooling curve shown in Figure S5. Upon further cooling to 28 °C (Figure 3C), the area fraction of the dark domains increased until the sample equilibrated to room temperature, after which a transition to a uniform bright phase was observed (Figure 3D). This uniform phase could potentially be a ripple phase, as reported by Kamal et al.^73^

In comparison, GUVs composed of DMPC with 30 mol % NAGS exhibited a homogeneous bright phase above 43 °C. Subsequent cooling to 39 °C induced the emergence of distinct dark domains. The differences in the shape and size of the emergent domains relative to those of DMPC-DPPC GUVs indicate that NAGS alterations in membrane organization extend beyond chain–chain phase separation. We highlight that DMPC-NAGS membranes have chain compositions identical to those of DMPC-DPPC membranes but differ in the headgroup chemistry. Once again, we expect that the charged nature of NAGS headgroups significantly impacts the lipid phase separation, in line with previous studies on mixtures of zwitterionic and charged lipids.^74^

As demonstrated in Video SI1, the diffusion of the dark domains on the bright background confirmed the fluid nature of the bright phase. Notably, the aggregation of the dark domains (Figure 3E, F) observed at ∼30 °C suggests that the domains are gel-like in nature, given that fluid domains tend to coalesce rather than aggregate. Upon further cooling to 26 °C, Figure 3G showed an increase in the roughness of the boundaries between the dark and bright phases. Along with this observation, as shown in Video SI2, the appearance of diffusive small dark domains within the bright phase indicates that the bright phase remained in the fluid phase. At room temperature (23 °C, Figure 3H), an intermediate brightness region emerged, suggesting a transitional phase with an intermediate packing density. This indicates the formation of a buffer zone between the ordered and disordered phases, thus lowering the energetic penalty associated with their coexistence. These observed morphological differences between DMPC-DPPC and DMPC-NAGS GUVs highlight the effect of headgroup chemistry on molecular phase separation.

**Figure 4:**
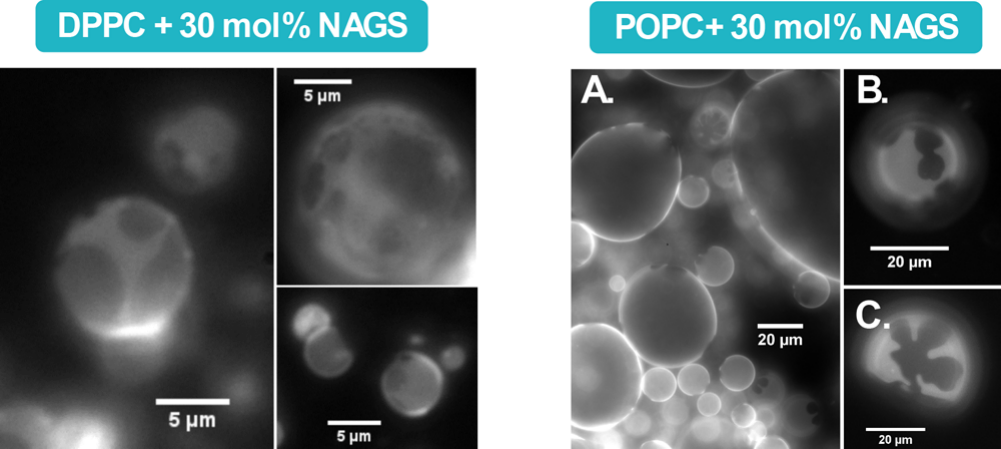
Epifluorescence images of PC lipid GUVs with 30 mol % NAGS, doped with DiI-C12 dye. (Left) Images show partial phase separation in DPPC-NAGS membranes as manifested in partially dark emergent domains. The GUVs were prepared at 60 °C and observed at 45 °C. (Right) POPC-NAGS membranes show different phase separations, with the emergence of structured domains upon cooling to room temperature. The GUVs were prepared at 40 °C and observed at: (A) 35 °C; (B) 27 °C; and (C) 22 °C.

The effect of NAGS headgroup chemistry on PC lipids was most evident in DPPC GUVs, as both compounds share a 16-carbon acyl chains. Unlike pure DPPC GUVs which exhibit no domain formation above their transition temperature, DPPC-NAGS GUVs displayed dark domains on a bright background upon cooling from 60 to approximately 45 °C (Figure 4). We conjecture that this is an outcome of the inhomogeneous distribution of DPPC and NAGS in the plane of the membrane, resulting in DPPC-rich and NAGS-rich domains. The less pronounced contrast of these domains, compared to DMPC-NAGS membranes, suggests a smaller difference in lipid order between DPPC- and NAGS-rich domains.

To further examine the effect of chain unsaturation on the phase separation, we performed analogous experiments on POPC-NAGS GUVs. As shown in Figure 4, dark domains emerged below 40 °C, similar to DMPC-NAGS samples. However, two key distinctions were noted. First, domain contrast was more pronounced in POPC-NAGS compared to DMPC-NAGS GUVs, likely due to POPC’s increased fluidity which facilitated more efficient dye inclusion into the disordered domains. Second, no further phase changes were observed as the sample was further cooled to room temperature, in contrast to the DMPC-NAGS membranes which exhibited additional phases upon cooling. Thermal undulations observed in the bright phase at room temperature suggest a fluid phase, as demonstrated in Video SI3.

In summary, our investigations revealed temperature-induced phase separation in DMPC, DPPC, and POPC membranes in the presence of NAGS. While all studies commenced with fully mixed membranes at high temperatures, a subsequent decrease in temperature resulted in the emergence, growth, and aggregation of ordered domains. This first-time study of fluid PC-NAGS GUVs reveals the formation of distinct domains at temperatures exceeding the main phase transition of pure DMPC, POPC, and DPPC. These findings emphasize the critical role of van der Waals interactions between the lipids and NAGS chains, coupled with PC-pyridinium headgroup interactions, driving the formation and properties of these domains. This highlights the potential of NAGS for controlling lipid distributions in fluid membranes. Future studies on different classes of GSs could elucidate additional effects that can result in different phases, enabling further capabilities in the NAGS tuning of membrane patterning.

## Conclusions

**Scheme 2:**
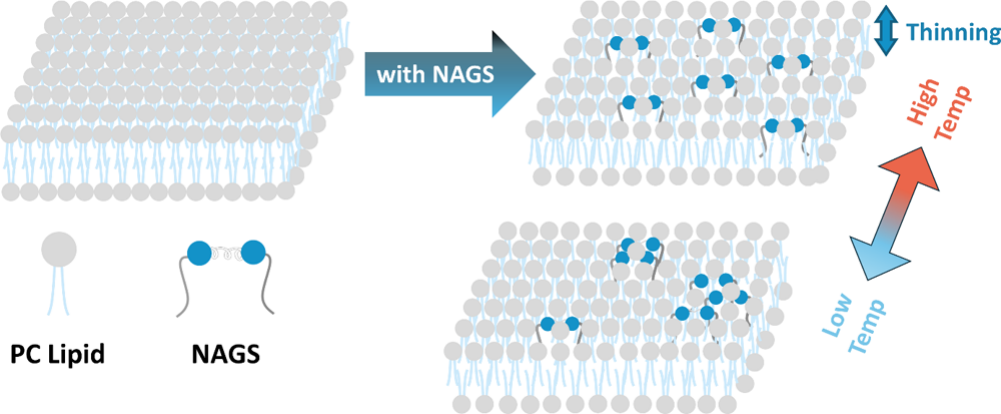
Illustration of the Effects of NAGS on Membrane Structure and Organization NAGS cause a reduction in membrane thickness and lipid packing density at high temperatures (50 °C), i.e., in the fully mixed fluid phase. Upon cooling to room temperature (22–25 °C), mixed lipid-NAGS membranes exhibit phase separation into NAGS-rich ordered domains and lipid-rich disordered domains.

This study explored how a novel surfactant, NAGS, interacts with various PC lipid membranes with identical or mismatched lipid chains. Specifically, our study considered DPPC membranes with identical lipid-NAGS chains, POPC membranes that differ in the length and unsaturation of one of the lipid chains, and DMPC membranes with shorter symmetric chains. The molecular modeling of lipid-NAGS pairs revealed attractive headgroup interactions between NAGS and all of the lipids. Interestingly, these interactions were strongest with DPPC membranes and weaker when the lipid tails did not perfectly match those of NAGS. Our SAXS studies revealed that the addition of NAGS to all studied membranes significantly influenced the packing of fluid PC membranes, causing a decrease in lipid packing and measurable membrane thinning above the transition temperature. DSC studies showed that high concentrations of NAGS (above 20 mol %) resulted in complex phase transitions, manifested in multiple transition peaks that are indicative of the emergence of heterogeneous domains that differ in thermal behavior and composition. Complementary epifluorescence studies corroborated that NAGS induced unconventional phase transitions that differed in shape, clustering, and composition from their analogous lipid–lipid mixtures. The compositional and structural model of PC-NAGS membranes, inferred from our experimental results, is illustrated in Scheme 2.

Overall, this study paves the way for future research on NAGS-based systems, particularly those pertaining to liposomal designs for drug delivery or other applications. Further investigation into how NAGS of different structures interact with different lipid mixtures could provide additional control in exploiting lipid/NAGS similarities and differences in tuning liposomal properties and functions.

## Supporting Information Available

Additional visual, experimental, and theoretical details on NAGS charge transfer complex in aqueous medium, dynamic light scattering (DLS) measurements of mixed PC-NAGS SUVs, differential scanning calorimetry (DSC) data analyses of mixed PC-NAGS membranes, small-angle X-ray scattering (SAXS) fitting parameters and data analysis (PDF)

## Supplementary Material

la4c03043_si_001.pdf

la4c03043_si_002.avi

la4c03043_si_003.avi

la4c03043_si_004.avi

## Abbreviations

ED: electron density
GS: gemini surfactant
NA: nicotinic acid
NAGS: nicotinic acid–based gemini surfactant
DPPC: 1,2-dipalmitoyl-*sn*-glycero-3-phosphocholine
DMPC: 1,2-dimyristoyl-*sn*-glycero-3-phosphocholine
POPC: 1-palmitoyl-2-oleoyl-*sn*-glycero-3-phosphocholine
POPS: 1-palmitoyl-2-oleoyl-*sn*-glycero-3-phospho-l-serine
DiI-C12: 1,1′-didodecyl-3,3,3′,3′-tetramethylindocarbocyanine perchlorate
SUV: small unilamellar vesicle
GUV: giant unilamellar vesicle
DLS: dynamic light scattering
DSC: differential scanning calorimetry
SAXS: small-angle X-ray scattering
TD-DFT: time-dependent density functional theory
*A*_L_: average area per lipid
*D*_B_: total bilayer thickness
*V*_H_: headgroup volume
*V*_C_: chain volume
