# Supplementary material for: Modification of Liposomal Properties by an Engineered Gemini Surfactant
Source: Langmuir. 2025 Jan 25;41(5):3042–52. doi: 10.1021/acs.langmuir.4c03043 (PMC11823631; doi:10.1021/acs.langmuir.4c03043)
Supplement: Supplementary file 1 — la4c03043_si_001.pdf [file la4c03043_si_001.pdf]

Supporting Information

Modification of Liposomal Properties by an Engineered Gemini Surfactant

Ala'a F. Eftaiha,[a,b,c] Buti Suryabrahmam,[b,c†] Nicholas B Morris,[b,c†] Abdussalam K. Qaroush,[d] Khaleel I. Assaf,[e] Dina M. Foudeh,[d] Suhad B. Hammad,[d] Rana Ashkar[b,c,f*]*

[a] Department of Chemistry, Faculty of Science, The Hashemite University, Zarqa 13133, Jordan

[b] Department of Physics, Virginia Tech, Blacksburg, VA 24061, USA

[c] Center for Soft Matter and Biological Physics, Virginia Tech, Blacksburg, VA 24061, USA

[d] Department of Chemistry, Faculty of Science, The University of Jordan, Amman 11942, Jordan

[e] Department of Chemistry, Faculty of Science, Al-Balqa Applied University, Al-Salt 19117, Jordan

[f] Macromolecular Innovation Institute, Virginia Tech, Blacksburg, VA 24061, USA

[†]These authors made an equal contribution to this study.

Corresponding authors: alaa.eftaiha@ hu.edu.jo; ashkar@vt.edu

Time-Dependent Density Functional Theory (TD-DFT) Calculations

TD-DFT calculations were used to simulate the UV-Visible absorption spectra and investigate the charge transfer complex phenomenon. Initially, geometry optimization was performed by the DFT method at M062X/6-31+G* level of theory using Gaussian 09 software.[1] The solvent effect used was considered using the universal continuum solvation model (SMD) for water.[2] Vibrational analysis ensured the local minima by the absence of imaginary frequencies. Excited states were calculated based on the optimized structures with the TD-DFT method using the same level of theory in water. GaussView 6.0.16 was used to visualize the results.

NAGS Charge Transfer Complex

Upon dissolution of NAGS in water at 60 °C, we observed an apparent change in solution color to yellow as shown in **Figure S1A**. This aligns with previous observation of yellowing in solutions of quaternary pyridinium iodides,[3–5] reporting that the absorption spectra of substituted 1-methylpyridinium iodides in aqueous solutions support the formation of charge-transfer complexes (CTCs). This phenomenon occurs due to electron transfer from the iodide anion to the π* orbital of the pyridinium ring. In accord with these observations, 4-alkyl-N-(benzyl)alkyl-pyridinium *bromide*,[6] as well as gemini pyridinium *iodide* compounds,[7] are expected to form CTCs. For 4-alkyl-N-(benzyl)alkyl-pyridinium *bromide* solutions, CTCs were observed in chloroform, water, and ethanol, whereas for gemini pyridinium *iodide* compounds, CTCs were formed in acetonitrile and DMSO as well.

Notably, the UV-visible absorption spectrum of the aqueous NAGS solution (**Figure S1B**) exhibited a peak at $\lambda_{\max}$ ~ 288 nm. Time-dependent density functional theory (TD-DFT)-calculated spectra (**Figure S1C&D**) demonstrated a discernible red shift in $\lambda_{\max}$ from 231 to 294 nm upon the addition of bromide anions. This observation correlates with the formation of CTC

discussed earlier. When incorporated into liposomal solutions of DPPC membranes, a similar yellowing of the suspensions was observed. The color intensity of DPPC/NAGS liposomal suspensions (**Figure S1A**) indicate a proportional increase with increasing NAGS concentration, featuring a maximum absorption at 288 nm (**Figure S1E**). This absorption pattern closely resembles the characteristic peak observed in the pure surfactant solution.

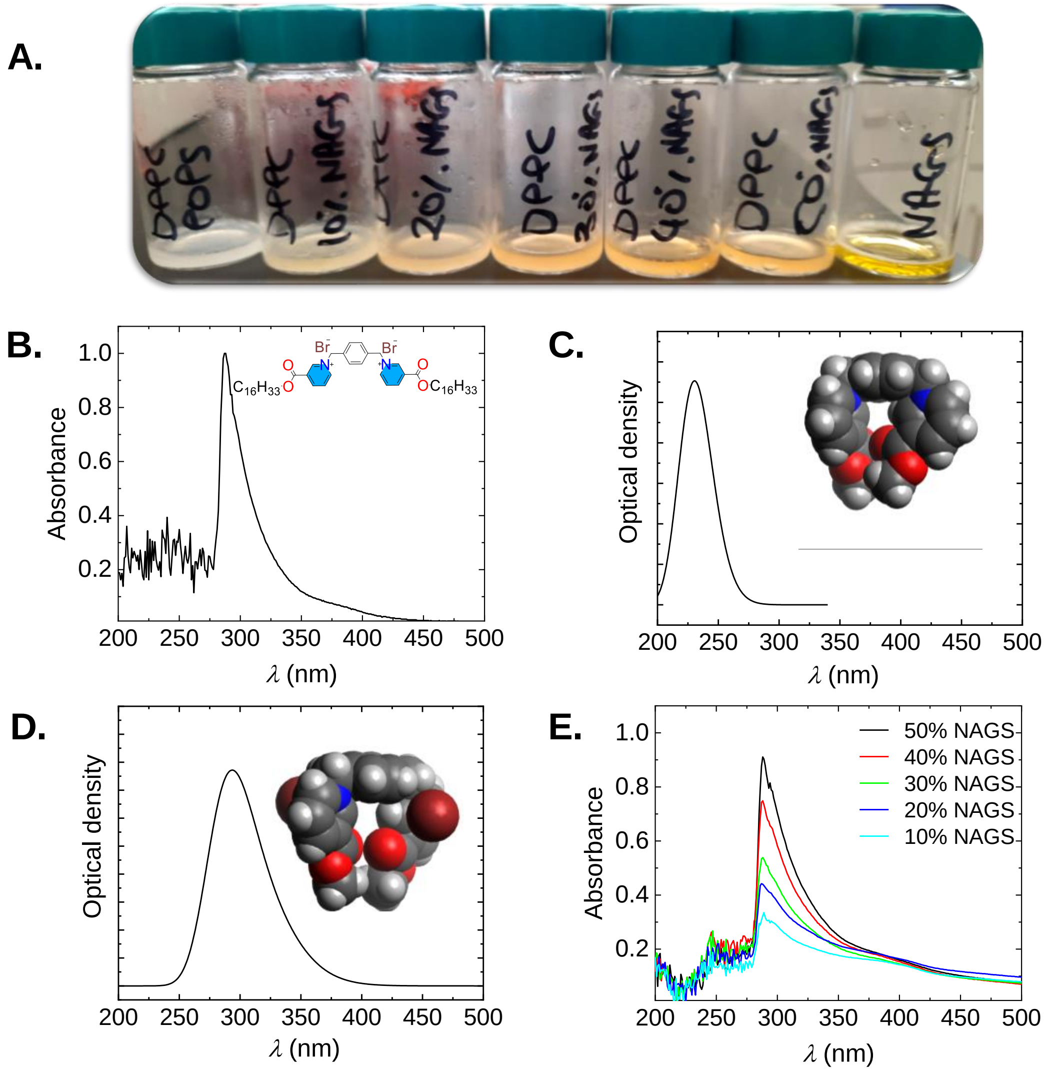

Figure S1. A. A photograph of aqueous suspensions of DPPC, NAGS, and their mixtures indicating deeper yellowing of the suspensions with increasing NAGS content from 10 to 50 mol%; **B.** Normalized UV-visible spectrum of NAGS aqueous solution indicating an absorbance peak at ~288 nm; **C**. and **D.** Calculated spectra at M062X/6-31+G* in water in the absence and presence of bromide anions, respectively. The addition of bromide ions causes a ~ 60 nm shift in the peak wavelength; **E.** UV-visible spectra of liposomal solutions of DPPC/NAGS, indicating an absorbance peak that is commensurate with the formation of charge-transfer complexes, in agreement with optical observations of color changes of the suspensions.

Size Analysis of Small Unilamellar Vesicles (SUVs)

Suspensions of lipid or lipid-NAGS vesicles were prepared using extrusion through polycarbonate filters with a pore diameter of 100 nm. To suppress multilamellarity, all vesicles were doped with 4 mol% of 1-palmitoyl-2-oleoyl-sn-glycero-3-phospho-L-serine (POPS).[8] To characterize the size distribution of the formed SUVs, we performed dynamic light scattering (DLS) studies of the vesicles hydrodynamic radius (R_H) and polydispersity index (PDI). To facilitate a meaningful comparison, the NAGS solution underwent extrusion as well, ensuring a systematic analysis. **Table S1** and **Figure S1** show the R_H values and size distribution profiles of SUVs of the examined lipids and their mixtures with NAGS. The mean radius of DPPC vesicles was found to be ~ 80 nm with a PDI < 0.1. The radii of the vesicles including NAGS ranged between 63 and 74 nm, with a PDI below 0.25, indicating relatively monodisperse SUVs. The same trend was observed for doped POPC vesicles up to 50 mol% NAGS. The extruded NAGS solution exhibited a smaller aggregate size (approximately 35 nm and PDI of 0.23) compared to the neat and mixed vesicles. Interestingly, the pure NAGS suspensions exhibited fundamentally different structural morphology, manifested in the appearance of weak diffraction peaks observed by SAXS (**Figure S3**).

As shown in **Table S1,** DLS measurements of DMPC SUVs and their mixed vesicles with NAGS exhibited an increase in the vesicle radius with increasing NAGS concentration. This could potentially arise from the softening of DMPC membranes with NAGS, enabling more deformations of the vesicles during the extrusion step and thus resulting in larger extruded vesicles. This is not unexpected for soft membranes. In this case, the mixing of DMPC and NAGS, which are characterized by nontrivial differences in the chain length, generates softer membrane in line with effects of chain mismatched cosurfactants.

Table S1. Hydrodynamic radius (R_H) and corresponding standard deviation (S.D.) of DPPC, POPC and DMPC liposomes and their mixtures with NAGS. All samples were doped with 4 mol% POPS. The NAGS solution was subjected to extrusion for comparison purposes.

	DPPC		POPC		DMPC	
Composition	R_H (nm)	S.D. (nm)	R_H (nm)	S.D. (nm)	R_H (nm)	S.D. (nm)
Lipid	161	3	125	2	122	2
NAGS 10%	140	4	127	4	130	1
NAGS 20%	147	1	136	2	128	7
NAGS 30%	131	2	136	2	146	3
NAGS 40%	127	6	137	3	232	14
NAGS 50%	126	1	138	5	273	37
NAGS	69	1				

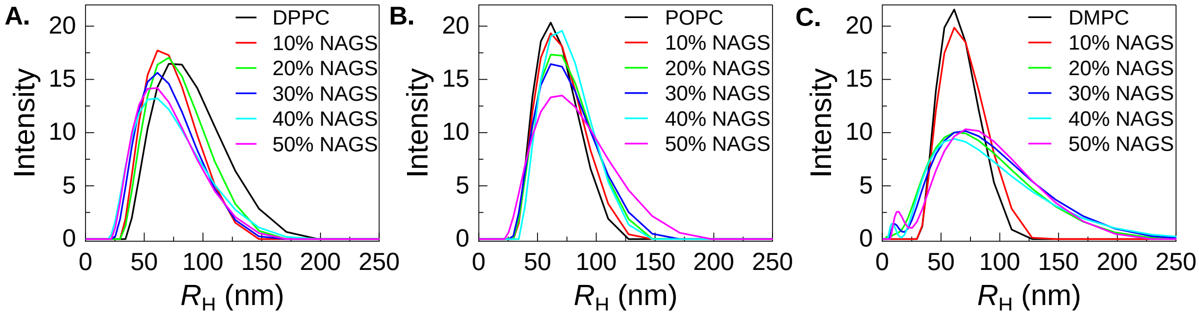

Figure S2. Dynamic light scattering measurements of vesicle suspensions reveal the size distribution of the vesicles in the form of the mean hydrodynamic radius, R_H. Data are shown for SUVs composed of: **A.** DPPC/NAGS, **B.** POPC/NAGS, and **C.** DMPC/NAGS. All samples were doped with 4 mol% POPS.

SAXS Measurements on Pure NAGS Suspensions

The scattering intensity profiles of pure NAGS, plotted against the scattering vector and measured at 25 and 50 °C are shown in **Figure S3**. We suggest that NAGS alone self-assembles into elongated micellar cylindrical structures or inverted hexagonal phase commonly found among various surfactant species. A complete investigation of supramolecular structure of NAGS is beyond the scope of this study but is reported here for completeness. Our analysis of the NAGS suspensions followed Frewein *et al.* [9] We found that the inner core has a much higher electron

density than the outer layer that determines the position of the electron rich headgroups. The length of the cylinders is estimated to be on the order of hundreds of nanometers, i.e., beyond the q-range of our SAXS data. In both low and high temperature regimes, a peak is observed at approximately $q = 0.12\ \text{Å}^{-1}$ corresponding to a spacing of about 60 Å for the (10) diffraction signal. At 25 °C, the (11) diffraction signal is clearly visible and higher order diffractions as predicted from theory[10] are much weaker but still present. This suggests enhanced clustering of inverse NAGS micelles at lower temperatures. Moreover, the signals exhibit an additional distinct feature manifested in a liftoff of the intensity minima relative to the background intensity (shown in the red shaded area in **Figure S3**) which is also minorly present in some of the lipid-NAGS mixtures as acknowledged in the text.

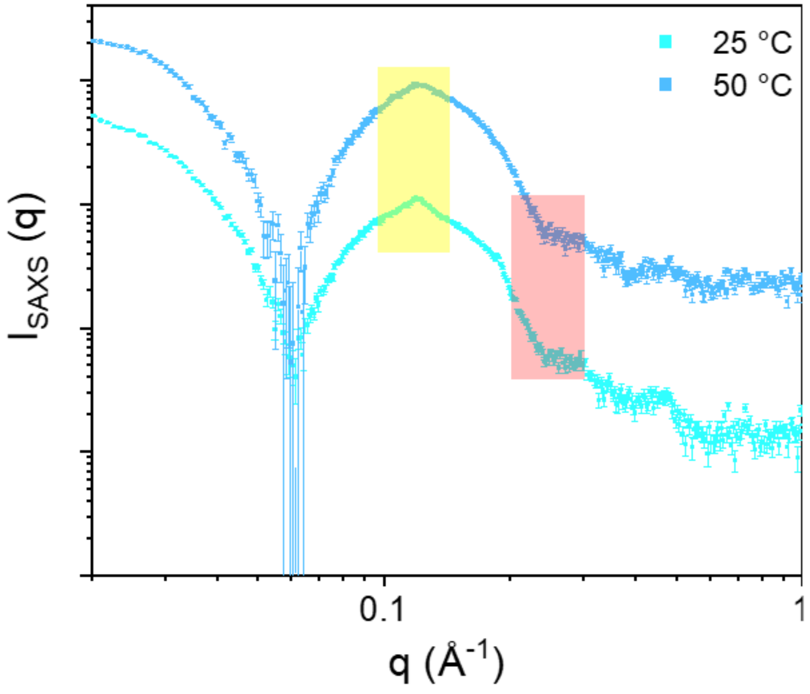

Figure S3. SAXS intensity profile for NAGS suspensions measured at 25 °C (light blue) and 50 °C (dark blue) indicate cylindrical micelle structures with a repeat distance of ~ 60 Å, indicated by the diffraction peak in the yellow highlighted region. The signals also show a liftoff in the intensity, relative to the background intensity, which most likely emanates from the form factor of elongated micellar assemblies.

SAXS Data Fitting of Lipid and Lipid-NAGS Membrane Structure

Data fitting was performed using a formalism adapted from the *EZ-SDP* model by Lewis-Laurent *et al.*[11] Briefly, membranes of lipids or lipid-NAGS mixtures were divided into 5 concentric spherical shells representing the outer headgroups, outer leaflet chains, spacing between the two leaflets, inner leaflet chains, and inner headgroups. The electron densities of each shell are determined from the X-ray scattering factors reported in **Table S2** as discussed below. The thicknesses of the shells are reported in **Table S3-S6**, as obtained from the best fits to the data. The details of the model are outlined below and the theoretical expressions were implemented into *SasView* for the fitting procedure.[12] Specifically, our model considers vesicles with a vesicle radius R_V with a headgroup shell thickness D_H and a corresponding headgroup electron density given by:

$$\rho_\mathrm{H} = \frac{b_\mathrm{H}}{A_\mathrm{L} D_\mathrm{H}} + \left(1 - \frac{V_\mathrm{H}}{A_\mathrm{L} D_\mathrm{H}}\right) \rho_\mathrm{S} \qquad \text{……….Eq. S1}$$

This includes contributions from the headgroup electrons dictating the headgroup X-ray scattering factor, b_H, as well as the water molecules surrounding the headgroup. Assuming an average area per lipid, A_L, the total volume of the headgroup region is then given by $A_\mathrm{L} D_\mathrm{H}$. By considering the molecular volume of the headgroup, V_H, this yields the remaining volume occupied by water, given by $A_\mathrm{L} D_\mathrm{H} - V_\mathrm{H}$. This volume is finally scaled by water's electron density at the corresponding temperature.

For the hydrocarbon shells representing the chain region, the electron density ρ_C is found by excluding the terminal methyl groups on the end of each chain, i.e., the total number of electrons for both chains in lipid or surfactant molecules minus the 18 electrons in the methyl groups. The number of electrons is then divided by the chain volume, V_C, minus the volume of the terminal methyl groups, V_T, from each of the opposing leaflets. This is given by:

$$\rho_C = \frac{b_C - 18}{V_C - 2V_T} \quad \text{..........Eq. S2}$$

The corresponding thickness D_C is given in terms of the chain volume and area per lipid as:

$$D_C = \frac{(V_C - 2V_T)}{A_L} \quad \text{.......... Eq. S3}$$

The electron density of the terminal methyl groups is given by $18/V_T$ with a shell thickness of $4V_T/A_L$, accounting for both methyl groups on both leaflets. V_T provides a measure of the distance between the leaflets and is not the true molecular volume of a methyl group. We note that the step-like SLD profile described in the 5-shell model above was convoluted with a Gaussian term with a standard deviation, σ, to account for thermal spreading across adjacent shells. Finally, a polydispersity to the vesicle radius was applied to accurately model the vesicle size distribution.

Here, we note that while the five-shell model for SAXS data fitting involves several fit parameters, some of these parameters have relatively narrow ranges set by the molecular structures. For example, the headgroup volume of PC lipids has been reported in previous studies, as shown in Table S2. Similarly, the headgroup volume of NAGS was calculated based on its atomic structure. These values were used to set a range for corresponding fit parameters, thus allowing for more reliable fitting of the rest of the structural observables reported in Tables S3-6.

Table S2. X-ray scattering factors of lipid or NAGS chains and headgroups, described by b_c and b_H respectively. The headgroup volumes are indicated by V_H.

Term†	**POPC**	**DPPC**	**DMPC**	**NAGS**
$\boldsymbol{b_C}$	256	242	210	258
$\boldsymbol{b_H}$	164	164	164	182
$\boldsymbol{V_H}$‡	330	330	330	307

† *The term for each mixture was calculated following additivity rule.*
‡ *V_H of the PC was obtained from simultaneous X-ray and neutron scattering measurements performed by Kučerka et al.*[13] *V_H of NAGS was evaluated based on van der Waals of its constituent atomic radii.*

Table S3. SAXS fitting parameters of DPPC SUVs and their NAGS mixtures measured at 50 °C. These include the average area per lipid (A_L), headgroup thickness (D_H), chain volume (V_C), volume of terminal methyl group (V_T), total bilayer thickness (D_B), and interfacial width (σ) between adjacent shells.

	**Composition**					
Parameter	**DPPC**	**10% NAGS**	**20% NAGS**	**30% NAGS**	**40% NAGS**	**50% NAGS**
$\boldsymbol{A_L}$ **(Å^2)**	62.6 ± 0.7	64.2 ± 1.0	65.9 ± 1.3	67.5 ± 1.5	70.3 ± 0.5	77.0 ± 0.5
$\boldsymbol{D_H}$ **(Å)**	8.6 ± 0.3	9.4 ± 0.3	9.4 ± 0.4	9.1 ± 0.4	9.4 ± 0.2	10.1 ± 0.2
$\boldsymbol{V_C}$ **(Å^3)**	880.3 ± 0.3	888.2 ± 0.4	892.6 ± 0.4	897.5 ± 0.5	901.0 ± 0.3	908.4 ± 0.4
$\boldsymbol{V_T}$ **(Å^3)**	58.6 ± 1.2	57.6 ± 1.7	55.7 ± 2.2	53.6 ± 2.3	51.4 ± 0.5	49.3 ± 0.6
$\boldsymbol{D_B}$ **(Å)**	38.7 ± 0.4	37.9 ± 0.6	37.0 ± 0.7	36.2 ± 0.8	34.8 ± 0.3	33.6 ± 0.2
$\boldsymbol{\sigma}$ **(Å)**	2.3	2.6	2.8	2.8	2.9	2.7

Table S4. SAXS fitting parameters of POPC SUVs and their NAGS mixtures measured at 50 °C. These include the average area per lipid (A_L), headgroup thickness (D_H), chain volume (V_C), volume of terminal methyl group (V_T), total bilayer thickness (D_B), and interfacial width (σ) between adjacent shells.

	**Composition**					
Parameter	**POPC**	**10% NAGS**	**20% NAGS**	**30% NAGS**	**40% NAGS**	**50% NAGS**
$\boldsymbol{A_L}$ **(Å^2)**	65.0 ± 1.3	66.3 ± 1.3	70.0 ± 1.7	76.7 ± 0.5	79.7 ± 0.2	80.4 ± 0.1
$\boldsymbol{D_H}$ **(Å)**	5.6 ± 0.4	5.9 ± 0.4	6.0 ± 0.5	6.3 ± 0.1	6.7 ± 0.1	6.7 ± 0.0
$\boldsymbol{V_c}$ **(Å^3)**	932.5 ± 0.9	937.3 ± 0.4	936.8 ± 0.6	938.0 ± 0.2	938.4 ± 0.5	940.6 ± 0.1
$\boldsymbol{V_T}$ **(Å^3)**	52.4 ± 1.4	50.3 ± 1.7	33.5 ± 2.5	11.7 ± 0.5	5.8 ± 0.1	4.5 ± 0.0
$\boldsymbol{D_B}$ **(Å)**	38.9 ± 0.9	38.1 ± 0.9	36.1 ± 1.0	32.9 ± 0.5	31.6 ± 0.3	31.3 ± 0.2
$\boldsymbol{\sigma}$ **(Å)**	2.8	3.2	3.9	4.5	4.6	4.6

Table S5. SAXS fitting parameters of DMPC SUVs and their NAGS mixtures measured at 50 °C. These include the average area per lipid (A_{L}), headgroup thickness (D_{H}), chain volume (V_{C}), volume of terminal methyl group (V_{T}), total bilayer thickness (D_{B}), and interfacial width (σ) between adjacent shells.

	**Composition**					
Parameter	**DMPC**	**10% NAGS**	**20% NAGS**	**30% NAGS**	**40% NAGS**	**50% NAGS**
$\boldsymbol{A_{\mathrm{L}}}$ **(Å²)**	63.3 ± 2.3	64.8 ± 1.4	67.8 ± 0.9	69.5 ± 0.7	76.6 ± 0.4	81.2 ± 0.4
$\boldsymbol{D_{\mathrm{H}}}$ **(Å)**	6.3 ± 0.6	7.1 ± 0.4	5.9 ± 0.3	6.8 ± 0.2	6.3 ± 0.1	6.8 ± 0.1
$\boldsymbol{V_{\mathrm{c}}}$ **(Å³)**	773.0 ± 0.3	794.8 ± 0.3	812.1 ± 0.1	838.9 ± 0.3	848.8 ± 0.3	867.4 ± 0.5
$\boldsymbol{V_{\mathrm{T}}}$ **(Å³)**	49.1 ± 3.7	49.4 ± 2.2	28.8 ± 1.3	17.7 ± 1.1	8.9 ± 0.1	7.4 ± 0.2
$\boldsymbol{D_{\mathrm{B}}}$ **(Å)**	34.9 ± 1.1	34.7 ± 0.9	33.6 ± 0.7	33.5 ± 0.6	30.5 ± 0.4	29.2 ± 0.4
$\boldsymbol{\sigma}$ **(Å)**	3.5	3.2	4.4	4.5	4.6	4.9

Table S6. SAXS fitting parameters of POPC SUVs and their NAGS mixtures measured at 25 °C. These include the average area per lipid (A_{L}), headgroup thickness (D_{H}), chain volume (V_{C}), volume of terminal methyl group (V_{T}), total bilayer thickness (D_{B}), and interfacial width (σ) between adjacent shells.

	**Composition**					
Parameter	**POPC**	**10% NAGS**	**20% NAGS**	**30% NAGS**	**40% NAGS**	**50% NAGS**
$\boldsymbol{A_{\mathrm{L}}}$ **(Å²)**	64.2 ± 0.6	64.8 ± 1.4	65.1 ± 1.8	67.6 ± 1.6	68.5 ± 0.7	69.7 ± 0.7
$\boldsymbol{D_{\mathrm{H}}}$ **(Å)**	6.9 ± 0.2	6.4 ± 0.9	6.2 ± 0.6	6.7 ± 0.5	6.8 ± 0.3	6.8 ± 0.2
$\boldsymbol{V_{\mathrm{c}}}$ **(Å³)**	920.6 ± 0.2	922.2 ± 0.5	923.1 ± 0.3	926.8 ± 0.6	925.2 ± 0.3	922.0 ± 0.2
$\boldsymbol{V_{\mathrm{T}}}$ **(Å³)**	51.6 ± 0.8	46.6 ± 3.7	40.5 ± 2.6	39.2 ± 2.7	37.8 ± 1.5	24.8 ± 1.8
$\boldsymbol{D_{\mathrm{B}}}$ **(Å)**	38.9 ± 0.4	38.6 ± 0.8	38.3 ± 1.0	37.0 ± 0.9	36.8 ± 0.4	35.6 ± 0.3
$\boldsymbol{\sigma}$ **(Å)**	2.5	3.7	3.9	3.7	4.0	4.2

SAXS Measurements of POPC-NAGS Membranes at Room-Temperature

It is worth noting that despite the fluid nature of POPC membranes at room temperature, fluorescence measurements (presented in the main text) showed domain heterogeneity within POPC/NAGS binary membranes in GUVs at 25 °C. Since the employed fitting model for SAXS measurements performed at 25 ° (**Figure S4**) assumes a homogeneous composition, the obtained fit parameters provide average values with the same trend in the fitting parameters, including A_L and D_B as observed at the 50 °C measurements.

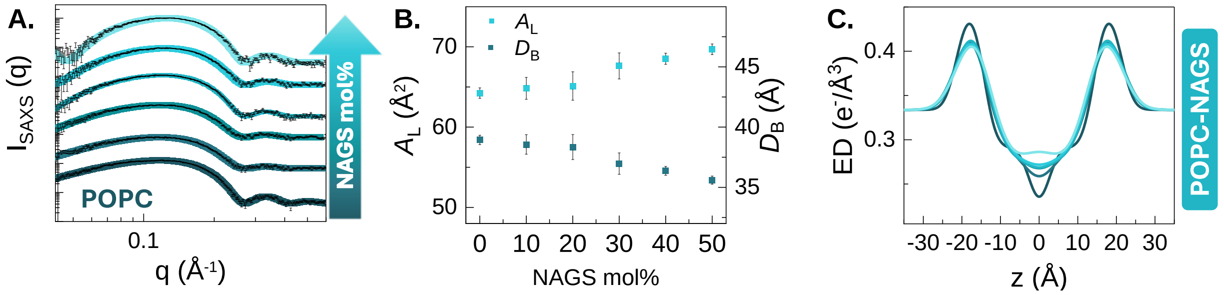

Figure S4. A. SAXS intensity profiles of SUV suspensions of POPC/NAGS measured at 25 °C. The NAGS content varied from 0 to 50 mol% in increments of 10 mol%; **B.** The area per lipid, A_L, and membrane thickness, D_B, obtained from SAXS data analysis indicate looser lipid packing and membrane thinning with increasing NAGS mol%; **C.** Electron density (ED) profiles reconstructed from SAXS data fitting show gradual changes in molecular arrangements along the membrane normal, with z = 0 indicating the membrane midplane. The complete set of SAXS fitted parameters is shown in **Tables S6.**

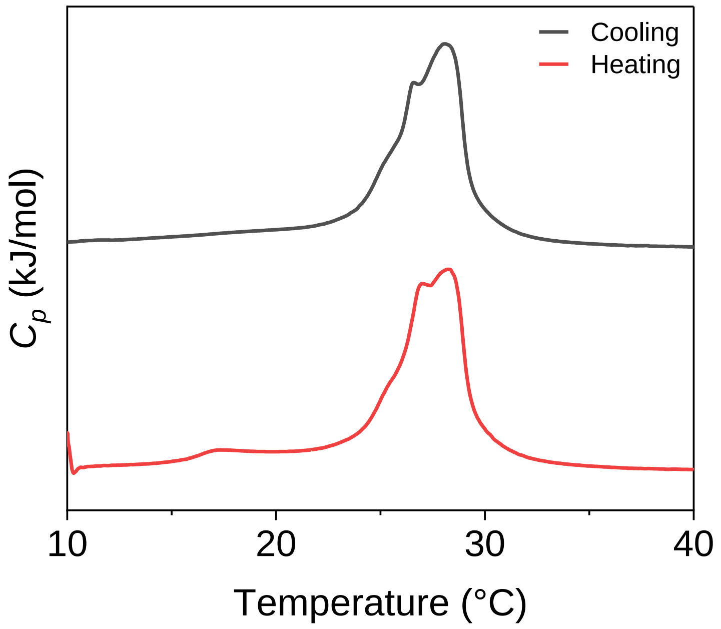

Figure S5. DSC thermograms (cooling, black trace; heating, red trace) for 7:3 DMPC-DPPC SUVs doped with 4 mol% of POPS. Prior to cooling, the sample was preheated to 60 °C and subsequently cooled down to 10 °C. The thermograms show the expected transitions of DMPC:DPPC mixtures, in agreement with other reports.

Video S1. Epifluorescence imaging shows diffusion and aggregation of ordered domains (dark) within a disordered membrane (bright) in GUVs composed of DMPC with 30 mol% NAGS at 30 °C.

Video S2. Epifluorescence imaging reveals the appearance of small diffusing ordered domains (dark) within the disordered phase (bright) in GUVs composed of DMPC with 30 mol% NAGS at 26 °C, indicating that the liposomal matrix (bright) remains in a fluid state.

Video S3. GUVs composed of POPC with 30 mol% NAGS at room temperature exhibit thermal undulations of the disordered liposomal matrix (bright).

References

(1) J. Frisch, G. W. Trucks, H. B. Schlegel, G. E. Scuseria, M. A. Robb, J. R. Cheeseman, G. Scalmani, V. Barone, B. Mennucci, G. A. Petersson, H. Nakatsuji, M. Caricato, X. Li, H. P. Hratchian, A. F. Izmaylov, J. Bloino, G. Zheng, J. L. Sonnenberg, M. Hada, M. Ehara, K. Toyota, R. Fukuda, J. Hasegawa, M. Ishida, T. Nakajima, Y. Honda, O. Kitao, H. Nakai, T. Vreven, J. A. Montgomery, Jr., J. E. Peralta, F. Ogliaro, M. Bearpark, J. J. Heyd, E. Brothers, K. N. Kudin, V. N. Staroverov, R. Kobayashi, J. Normand, K. Raghavachari, A. Rendell, J. C. Burant, S. S. Iyengar, J. Tomasi, M. Cossi, N. Rega, J. M. Millam, M. Klene, J. E. Knox, J. B. Cross, V. Bakken, C. Adamo, J. Jaramillo, R. Gomperts, R. E. Stratmann, O. Yazyev, A. J. Austin, R. Cammi, C. Pomelli, J. W. Ochterski, R. L. Martin, K. Morokuma, V. G. Zakrzewski, G. A. Voth, P. Salvador, J. J. Dannenberg, S. Dapprich, A. D. Daniels, O. Farkas, J. B. Foresman, J. V. Ortiz, J. Cioslowski, and D. J. Fox. Gaussian 09, 2009.

(2) Marenich, A. V.; Cramer, C. J.; Truhlar, D. G. Universal Solvation Model Based on Solute Electron Density and on a Continuum Model of the Solvent Defined by the Bulk Dielectric Constant and Atomic Surface Tensions. *J. Phys. Chem. B* **2009**, *113* (18), 6378–6396. https://doi.org/10.1021/jp810292n.

(3) Kosower, E. M. Additions to Pyridinium Rings. I. 1-Methylpyridinium Iodide. *J. Am. Chem. Soc.* **1955**, *77* (14), 3883–3885. https://doi.org/10.1021/ja01619a060.

(4) Kosower, E. M.; Klinedinst, P. E. Jr. Additions to Pyridinium Rings. II. Charge-Transfer Complexes as Intermediates. *J. Am. Chem. Soc.* **1956**, *78* (14), 3493–3497. https://doi.org/10.1021/ja01595a061.

(5) Kosower, E. M. Additions to Pyridinium Rings. III. Chemical and Biochemical Implications of Charge-Transfer Complex Intermediates. *J. Am. Chem. Soc.* **1956**, *78* (14), 3497–3501. https://doi.org/10.1021/ja01595a062.

(6) Boucher, E. A.; Mollett, C. C. Coloured and Colourless Charge-Transfer Complexes of Small and Polymeric Quaternary Pyridinium Bromides. *J. Chem. Soc. Faraday Trans. 1* **1982**, *78* (5), 1401–1404. https://doi.org/10.1039/F19827801401.

(7) Rodrigues de Carvalho, F.; da Silva, F.; de Lima, R.; Correia Bellotto, A.; de Souza, V. R.; Caetano, W.; Politi, M. J.; Hioka, N.; Coutinho, K. Spectrophotometric Studies of Charge-Transfer Complexes Formed with Ions N,N'-Alkyldiyl-Bis(Pyridinium) Derivatives and Iodide. *Spectrochim. Acta. A. Mol. Biomol. Spectrosc.* **2022**, *268*, 120664. https://doi.org/10.1016/j.saa.2021.120664.

(8) Scott, H. L.; Skinkle, A.; Kelley, E. G.; Waxham, M. N.; Levental, I.; Heberle, F. A. On the Mechanism of Bilayer Separation by Extrusion, or Why Your LUVs Are Not Really Unilamellar. *Biophys. J.* **2019**, *117* (8), 1381–1386. https://doi.org/10.1016/j.bpj.2019.09.006.

(9) Frewein, M. P. K.; Rumetshofer, M.; Pabst, G. Global Small-Angle Scattering Data Analysis of Inverted Hexagonal Phases. *J. Appl. Crystallogr.* **2019**, *52* (2), 403–414. https://doi.org/10.1107/S1600576719002760.

(10) Oster, G.; Riley, D. P. Scattering from Cylindrically Symmetric Systems. *Acta Crystallogr.* **1952**, *5* (2), 272–276. https://doi.org/10.1107/S0365110X5200071X.

(11) Lewis-Laurent, A.; Doktorova, M.; Heberle, F. A.; Marquardt, D. Vesicle Viewer: Online Visualization and Analysis of Small-Angle Scattering from Lipid Vesicles. *Biophys. J.* **2021**, *120* (21), 4639–4648. https://doi.org/10.1016/j.bpj.2021.09.018.

(12) Guinier, A.; Fournet, G. *Small-Angle Scattering of X-Rays*; John Wiley & Sons Inc., New York, 1955.

(13) Kučerka, N.; Nieh, M.-P.; Katsaras, J. Fluid Phase Lipid Areas and Bilayer Thicknesses of Commonly Used Phosphatidylcholines as a Function of Temperature. *Biochim. Biophys. Acta BBA - Biomembr.* **2011**, *1808* (11), 2761–2771. https://doi.org/10.1016/j.bbamem.2011.07.022.
